# Construction and validation of machine learning algorithm for predicting depression among home-quarantined individuals during the large-scale COVID-19 outbreak: based on Adaboost model

**DOI:** 10.1186/s40359-024-01696-8

**Published:** 2024-04-24

**Authors:** Yiwei Zhou, Zejie Zhang, Qin Li, Guangyun Mao, Zumu Zhou

**Affiliations:** 1 Business School, University of Shanghai for Science and Technology, 200093 Shanghai, China; 2 School of Intelligent Emergency Management, University of Shanghai for Science and Technology, 200093 Shanghai, China; 3 Smart Urban Mobility Institute, University of Shanghai for Science and Technology, 200093 Shanghai, China; 4 Wenzhou Center for Disease Control and Prevention, 325000 Wenzhou, China; 5 The Affiliated Kangning Hospital of Wenzhou Medical University Zhejiang Provincial Clinical Research Center for Mental Disorders, 325007 Wenzhou, China; 6 Department of Preventive Medicine, School of Public Health, Wenzhou Medical University, 325035 Wenzhou, China

**Keywords:** COVID-19, Machine learning, Home-quarantined individuals, Adaboost Algorithm

## Abstract

**Objectives:**

COVID-19 epidemics often lead to elevated levels of depression. To accurately identify and predict depression levels in home-quarantined individuals during a COVID-19 epidemic, this study constructed a depression prediction model based on multiple machine learning algorithms and validated its effectiveness.

**Methods:**

A cross-sectional method was used to examine the depression status of individuals quarantined at home during the epidemic via the network. Characteristics included variables on sociodemographics, COVID-19 and its prevention and control measures, impact on life, work, health and economy after the city was sealed off, and PHQ-9 scale scores. The home-quarantined subjects were randomly divided into training set and validation set according to the ratio of 7:3, and the performance of different machine learning models were compared by 10-fold cross-validation, and the model algorithm with the best performance was selected from 15 models to construct and validate the depression prediction model for home-quarantined subjects. The validity of different models was compared based on accuracy, precision, receiver operating characteristic (ROC) curve, and area under the ROC curve (AUC), and the best model suitable for the data framework of this study was identified.

**Results:**

The prevalence of depression among home-quarantined individuals during the epidemic was 31.66% (202/638), and the constructed Adaboost depression prediction model had an ACC of 0.7917, an accuracy of 0.7180, and an AUC of 0.7803, which was better than the other 15 models on the combination of various performance measures. In the validation sets, the AUC was greater than 0.83.

**Conclusions:**

The Adaboost machine learning algorithm developed in this study can be used to construct a depression prediction model for home-quarantined individuals that has better machine learning performance, as well as high effectiveness, robustness, and generalizability.

**Supplementary Information:**

The online version contains supplementary material available at 10.1186/s40359-024-01696-8.

## Background

The global coronavirus disease (COVID-19) pandemic caused by the SARS-CoV-2 had a severe social and economic impact, resulting in many cases of morbidity and mortality, as well as extensive negative effects on people’s mental health. In the early days of the large-scale COVID-19 outbreak in Shanghai in the first half of 2022, the local government took strict preventive and control measures to contain the epidemic. The implementation of a prolonged city closure strategy, restricting residents from going out, and the prolonged quarantine at home, as well as the changes in the economy, society, and life after the implementation of the city closure measures, were prone to cause psychological distress among those living in home quarantine, and experienced an increased level of psychological stress, anxiety, and depression [1–2].

Depression is a common mental disorder that is often accompanied by somatic symptoms, mainly fatigue, pain or sleep disturbances [3–4]. Depressed mood may or may not be present. Characterized by persistent sadness, hopelessness and loss of interest in once enjoyable activities, depression is a mental illness that affects millions of people worldwide. Depression is a heterogeneous disorder with a variable course, inconsistent response to treatment and no defined mechanism [5–6]. Depression is now widely recognized as a complex multifactorial disorder characterized by affective, cognitive and psychosocial symptoms [7]. Additionally, depression is a major public health problem, a leading cause of disability, morbidity, hospital admissions and excess mortality, and carries a high risk of suicide [8–11]. WHO estimates that 3.8% of the population suffers from depression, including 5% of adults (4% of men and 6% of women) and 5.7% of adults over 60 years of age. Approximately 280 million people worldwide suffer from depression. Depression can lead to suicide, and more than 700,000 people die by suicide each year as a result of depression [12].

In recent years, machine learning models have been widely used in various clinical aspects. A large number of machine learning algorithms, such as decision tree (DT), random forest (RF), K nearest neighbors (KNN), gradient enhancement (GB), light gradient enhancement machine (LightGBM), and extreme gradient enhancement (XGBoost), artificial neural networks (ANN), discriminant analysis, and regression analysis, etc., have been applied to the diagnosis and treatment of clinical diseases [13–17]. In addition, many researchers use machine learning models to predict depression in patients with chronic diseases such as hypertension, diabetes, stroke, cancer, etc [18–23]. Byeon et al. [24] used stacking ensemble machine technology to analyze epidemiological survey data on depression among elderly women living alone in South Korea, explored the major risk factors for depression, and developed a nomogram to help primary care physicians easily interpret high-risk populations for depression in primary care settings. Three different ensemble learning classifiers, Random Forest (RF), Extreme Gradient Boosting (XGBoost), and Light Gradient Boosting Machine (LightGBM), were used to analyze the mental health data of 15,173 older adults and to diagnose and identify mental health disorders such as depression in older adults, and the ensemble learning classifiers were evaluated, and it was found that all three classifiers achieved good prediction results, with the LightGBM algorithm having a higher accuracy rate than the Random Forest algorithm and the XGBoost algorithm [25]. Li et al. [26] used the XGBoost machine learning algorithm for monitoring and behavioral analysis of depression among men who have sex with men (MSM) using online social network data, and the results showed that this algorithm can help identify high-risk populations who are in the early stages of depression, which can contribute to further diagnosis.

In recent years, machine learning algorithms have also been used to predict and diagnose depression in various populations under COVID-19 pandemic conditions [20, 27–28]. Healthcare workers are an important high-risk group for mental health problems during the COVID-19 pandemic. Irfan et al. [29] used various machine learning algorithms such as decision tree (DT), random forest (RF), K nearest neighbor classification algorithm (KNN), gradient based augmentation (GB), light gradient boosting machine (LightGBM), and extreme gradient boosting machine (XGBoost) to predict the impact of COVID-19 epidemic on the psychological status of frontline healthcare workers in Saudi Arabia, suggesting the value of machine learning algorithms in predicting the mental health of healthcare workers during disease epidemics. Portugal et al. [30] collected data through a web-based survey and used a machine learning algorithm linear ε-insensitive support vector machine (ε-SVM) to predict the depressive symptoms of healthcare workers. The results were also satisfactory. Qasrawi et al. [31] evaluated the performance of seven machine learning algorithms for predicting symptoms of depression and anxiety during the COVID-19 pandemic in July-December 2020. They used machine learning (ML) models to predict depression and anxiety in women from Arab countries. The results showed the ability of ML models to predict maternal depression and anxiety, and demonstrated that machine learning models were effective tools for identifying and predicting relevant risk factors affecting maternal mental health. It was also shown that the gradient boosting algorithm (GB) and random forest (RF) models outperformed other machine learning algorithms. Ren et al. [32] used Akaike Information Criterion (AIC) and multivariate logistic regression to evaluate the influencing factors of COVID-19 on college students’ anxiety and depression, and the results showed that the extent to which family economic status was affected by COVID-19 had a significant influence on anxiety or depression. During the COVID-19 epidemic in Brazil and Spain, Simjanoski et al. [33] analyzed the lifestyle of 22,562 subjects using the ElasticNet algorithm, random forest, and XGBoost, and concluded that certain lifestyle factors could serve as predictors of depression.

Although machine learning techniques have been widely used in the diagnosis and treatment of clinical diseases, in the prediction of depression in patients with multiple chronic diseases, and in the prediction of factors related to depression in various populations during the COVID-19 epidemic, machine learning techniques for predicting depression in quarantined individuals during the COVID-19 epidemic are rare. Currently, no reports of the Adaboost algorithm for predicting depression in home-quarantined populations during a large-scale COVID-19 outbreak, which was characterized by a wide treach, a large number of people affected and a long quarantine period in the first half of 2022, have been retrieved from databases such as Pubmed.

The purpose of this study is to select the most optimal machine learning algorithm to construct and validate a model for predicting depression in home-quarantined individuals during the COVID-19 epidemic. This study is based on survey data on the depression status of these individuals during the early stage of the COVID-19 outbreak in Shanghai, and the best Adaboost algorithm was selected from 15 machine learning algorithms to predict the depression status of home-quarantined people during the COVID-19 epidemic. This provides a timely prediction of depression in similar events in the future and a timely warning for individuals at high risk of depression, providing a scientific basis for decision-making by the government and relevant departments.

## Subjects and methods

### Participants

The study participants were individuals who were quarantined at home during the COVID-19 epidemic in Shanghai in the first half of 2022. From April 20 to May 20, 2022, the questionnaire was sent to respondents via WeChat using the questionnaire star platform. The WeChat link was then sent to subsequent respondents using the snowball method. Respondents independently completed the questionnaire online upon receipt of the survey request and submitted it online upon completion.

### Survey content

The cross-sectional survey method was used in this study. The questionnaire was designed by the researcher herself. The questionnaires in this study were used to obtain data via the Internet, and if participants filled out incomplete questionnaires, they could not submit them via the Internet to avoid missing values. In this study, we preprocessed the data before building the machine learning model to check for obvious logical errors, duplicate values, missing values, etc. The content of the survey mainly included three parts: The first part was general demographic characteristics, including gender, age, professional title, occupation, monthly income, marital status, and so on. The second part was related to the COVID-19 epidemic and depression factors, including quarantine days, nucleic acid testing, knowledge of COVID-19, vaccine dose(s), and worries about epidemic control, unemployment of self and family members, health of self and family members, lack of daily necessities, shortage of food during the epidemic, worries about the effectiveness of prevention and control measures, children’s schooling, regular work affected, and bad mood during quarantine. The third part was the Patient Health Questionnaire 9 (PHQ-9).

### Survey instrument

The PHQ-9 is a brief self-report depression questionnaire that can be used to screen for depressive disorders and to assess the severity of depression. The scale can be used to find out how long the patient has suffered from 9 problems, including low mood, decreased interest, sleep disturbance, lack of energy, eating disturbance, low self-esteem, difficulty concentrating, irritability, and negative perceptions, in the past 2 weeks. The PHQ-9 consists of 9 items, and the scores for each item are as follows: 0 = not at all; 1 = a few days; 2 = more than half the days; and 3 = almost every day. The scale has a total score of 27, with the higher the score, the greater the likelihood of having a depressive disorder. Scores of 0–4 are considered no depressive symptoms, 5–9 are considered mild mood, 10–14 are considered moderate, and 15 or more are considered severe; with a total score of ≥ 10 as the cutoff for possible depressive disorder. The scale has good validity and reliability.

### Statistical methods

In this study, Python (version 3.8.8) and PyCaret (version 2.3.10) were used to construct a machine learning pipeline in the home-quarantined population database. During the model building process, sociodemographic characteristics such as age, gender, educational level, occupation, professional title, marital status, and income were combined, and feature selection was performed using the RFECV algorithm, which ultimately determined the construction of a model containing 22 features. We compare the performance of 15 machine learning models, including Ridge Classifier, Light Gradient Boosting Machine, and Adaboost, and identify the optimal model through 10-fold cross-validation. After tuning the models, the data set is divided into training and validation sets at a ratio of 7:3. Then, the models are constructed based on the training set, and performance metrics such as accuracy, AUC, recall, etc. are comprehensively tested on the validation set to further evaluate the performance of the models.

The performance of classification-based algorithms can be evaluated based on accuracy, precision, recall, F1 score, and AUC [34]. The machine learning performance metrics were calculated using the following methods: Accuracy = (TP + TN) / (TP + TN + FP + FN); Precision = TP / (TP + FP); Recall = TP / (TP + FN); and F1 score = 2 / [(1/recall) + (1/precision)]. FN is the false negative rate, FP is the false positive rate, TN is the true negative rate, TP is the true positive rate.

### The flowchart of the model construction

**Fig. 1:**
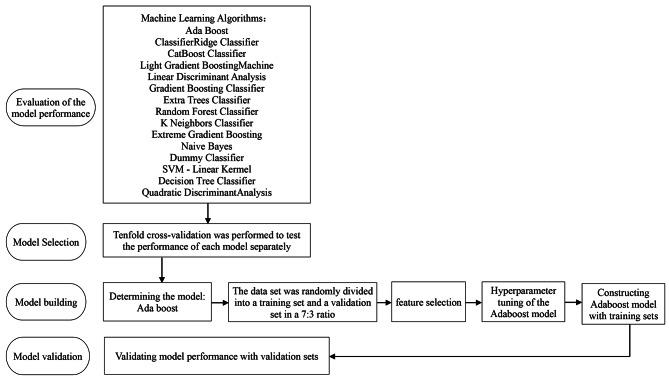
Flowchart of the Adaboost algorithm

In our study, the steps in the modeling process include evaluation of the model performance, model selection, model building, and model validation, see Fig. 1.

### Ethical review

This study was reviewed and approved by the medical ethics committee of our hospital (YSSL2022008). The questionnaire includes the purpose and significance of this survey, which was explained to the respondents before the survey and their informed consent was obtained. If you do not agree, you cannot complete the questionnaire.

## Results

### Sociodemographic characteristics

The obvious logical errors, duplicate values, missing values, etc. were not found in the data from our study. In reviewing the data from 649 participants, we found that 11 participants answered the questions in less than 20 s, which we considered invalid data and therefore excluded from this study. A total of 638 participants were enrolled in the study, of whom 240 were male; 242 were ≤ 29 years old; 200 had an industrial or commercial occupation; 529 had no or a junior professional title; 369 were married; and 223 had an income of ≥ 10,000 RMB monthly. 280 were quarantined for 3 to 4 weeks; 106 had no or little knowledge of the COVID-19; 16 were nucleic acid test positive; 65 were not vaccinated or not fully vaccinated; 300 worried about the difficulty of epidemic prevention and control; 510 worried about their own or their family members’ unemployment; 392 worried about their own or their family members’ health; 371 worried about the inability to secure daily necessities; 322 worried about the inability to secure food; 302 worried about the effect of prevention and control measures; 511 worried about schooling for children; 466 worried about the performance of their regular work; 407 were in a bad mood during the quarantine; 481 worried about traffic halt, see Table 1. The prevalence of depression symptoms was 31.66% among the home-quarantined subjects in our study.

**Table 1:** Demographic characteristics and depression among home quarantined participants

Characteristics	subsamples	Low depression (๤ 9 scores)		High depression (≥ 10 scores)	
		n	%	n	%
**Gender**					
Male	240	168	70.00	72	30.00
Female	398	268	67.34	130	32.66
**Age (years)**					
≤ 29	242	141	58.26	101	41.74
30–49	248	182	73.39	66	26.61
≥ 50	148	113	76.35	35	23.65
**Occupation**					
Industry or business	200	138	69.00	62	31.00
Health or education	133	97	72.93	36	27.07
Administer	145	97	66.90	48	33.10
Agriculture or forestry etc.,	160	104	65.00	56	35.00
**Professional title**					
Low	529	354	66.92	175	33.08
Middle	81	61	75.31	20	24.69
High	28	21	75.00	7	25.00
**Marital status**					
Married	369	266	72.09	103	27.91
Single	269	170	63.20	99	36.80
**Income/month (RMB)**					
≤ 1,999	162	94	58.02	68	41.98
2,000–4,999	106	61	57.55	45	42.45
5,000–9,999	147	103	70.07	44	29.93
≥ 10,000	223	178	79.82	45	20.18
**Quarantined for (weeks)**					
≤ 2	35	26	74.29	9	25.71
3–4	280	188	67.14	92	32.86
5–6	229	159	69.43	70	30.57
≥ 7	94	63	67.02	31	32.98
**Knowledge about COVID-19**					
No or little	106	69	65.09	37	34.91
Some	228	137	60.09	91	39.91
Very knowledgeable	106	32	30.19	74	69.81
**Nucleic acid test**					
Negative	622	429	68.97	193	31.03
Positive	16	7	43.75	9	56.25
**Vaccination**					
No or uncompleted	65	42	64.62	23	35.38
Completed	181	134	74.03	47	25.97
Booster vaccination	392	260	66.33	132	33.67
**Worry about uncertain duration of the pandemic**					
Yes	300	227	75.67	73	24.33
No	338	209	61.83	129	38.17
**Worry about unemployment of oneself or family members**					
Yes	510	383	75.10	127	24.90
No	128	53	41.41	75	58.59
**Worry about health of oneself or family members**					
Yes	392	286	72.96	106	27.04
No	246	150	60.98	96	39.02
**Worry about lack of daily necessities**					
Yes	371	284	76.55	87	23.45
No	267	152	56.93	115	43.07
**Worry about lack of food supply**					
Yes	322	243	75.47	79	24.53
No	316	193	61.08	123	38.92
**Worry about the effectiveness of countermeasures**					
Yes	302	229	75.83	73	24.17
No	336	207	61.61	129	38.39
**Worry about schooling for children**					
Yes	511	363	71.04	148	28.96
No	127	73	57.48	54	42.52
**Worry about routine work affected**					
Yes	466	358	76.82	108	23.18
No	172	78	45.35	94	54.65
**Bad mood during quarantine**					
Yes	407	340	83.54	67	16.46
No	231	96	41.56	135	58.44
**Worry about traffic halt**					
Yes	481	358	74.43	123	25.57
No	157	78	49.68	79	50.32

### Model algorithm selection

First, we constructed 15 classification machine learning models and evaluated their effectiveness in terms of accuracy, AUC, recall, precision, F1-score, Kappa value and MCC. The results showed that the Adaboost model had more excellent classification ability than other 14 models in terms of accuracy (0.7894), F1-score (0.6046), Kappa (0.4656), and MCC (0.4799); the AUC (0.7767), recall (0.5484), and precision (0.7056) also remain excellent; the detailed performance of each machine learning model was shown in Table 2. Therefore, the Adaboost algorithm was selected for further analysis in this study.

**Table 2:** Performance evaluation of each machine learning model with default parameters

model	Accuracy	AUC	Recall	Precision	F1-score	Kappa	MCC
Ada Boost Classifier	0.7894	0.7767	0.5484	0.7056	0.6049	0.4656	0.4799
Ridge Classifier	0.7895	0.0000	0.5110	0.7214	0.5891	0.4536	0.4712
CatBoost Classifier	0.7784	0.7672	0.4374	0.7160	0.5345	0.4023	0.4274
Light Gradient Boosting Machine	0.7764	0.7498	0.5352	0.6486	0.5852	0.4347	0.4390
Linear Discriminant Analysis	0.7813	0.5407	0.6580	0.6580	0.5878	0.4369	0.4445
Gradient Boosting Classifier	0.7716	0.7437	0.4956	0.6523	0.5618	0.4118	0.4196
Extra Trees Classifier	0.7627	0.7613	0.4143	0.6585	0.5048	0.3619	0.3797
Random Forest Classifier	0.7626	0.7652	0.3978	0.6753	0.4952	0.3554	0.3788
K Neighbors Classifier	0.7512	0.6783	0.3533	0.6517	0.4561	0.3138	0.3391
Extreme Gradient Boosting	0.7403	0.7378	0.4511	0.5901	0.5054	0.3350	0.3436
Naive Bayes	0.7266	0.7537	0.6297	0.5393	0.5785	0.3796	0.3833
Dummy Classifier	0.7018	0.5000	0.0000	0.0000	0.0000	0.0000	0.0000
SVM - Linear Kermel	0.6886	0.0000	0.5066	0.5422	0.4605	0.2624	0.2920
Decision Tree Classifier	0.6371	0.5849	0.4445	0.4032	0.4181	0.1582	0.1603
Quadratic Discriminant Analysis	0.5670	0.5271	0.4154	0.3138	0.3382	0.0406	0.0426

### Construction of Adaboost machine learning model

#### Order of importance of characteristics

**Fig. 2:**
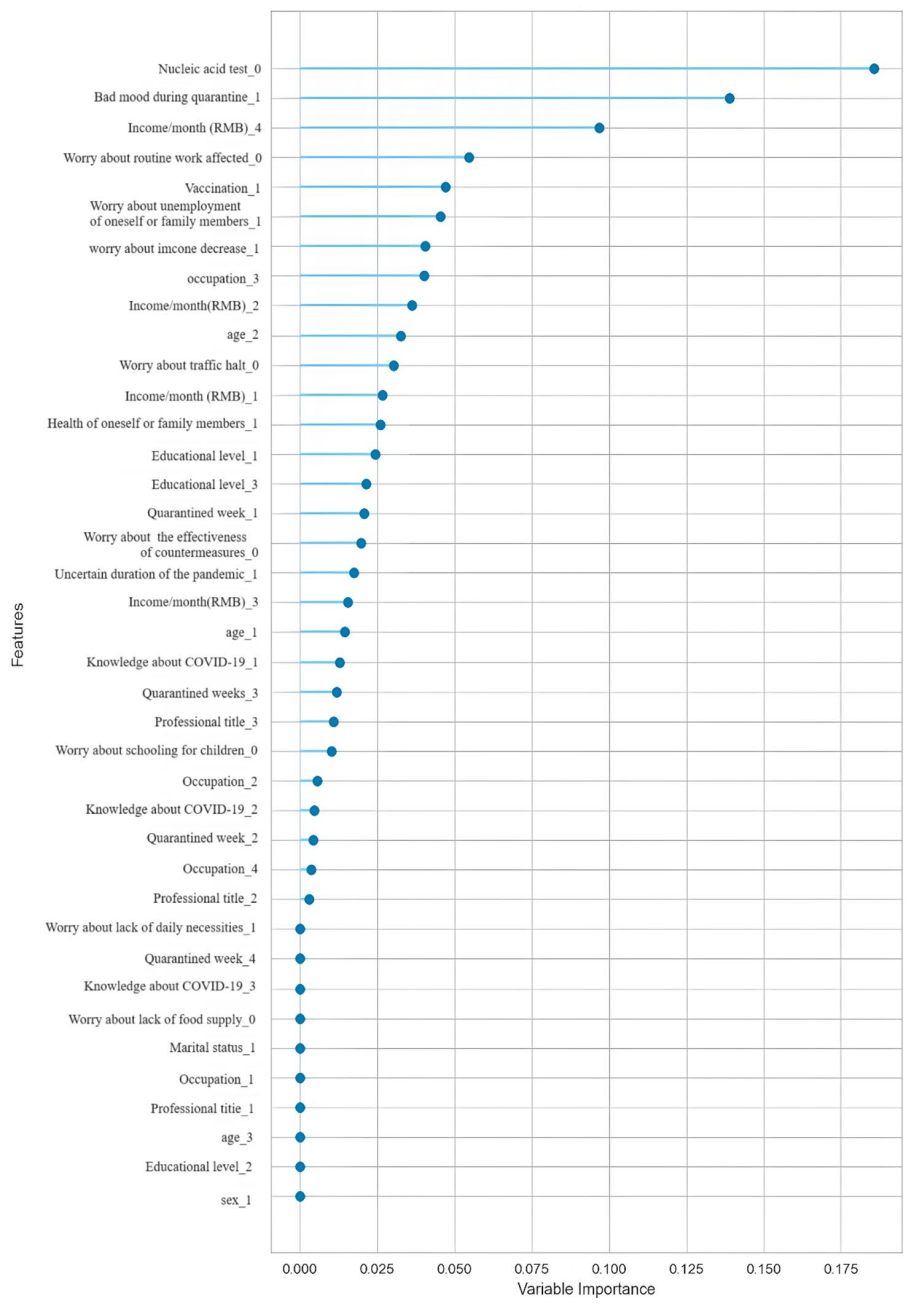
Order of the importance of various features

The order of importance of each sociodemographic, COVID-19 outbreak, and other mental mental characteristics contributing to the depression symptoms in this study was shown in Fig. 2.

### *Feature selection*

**Fig. 3:**
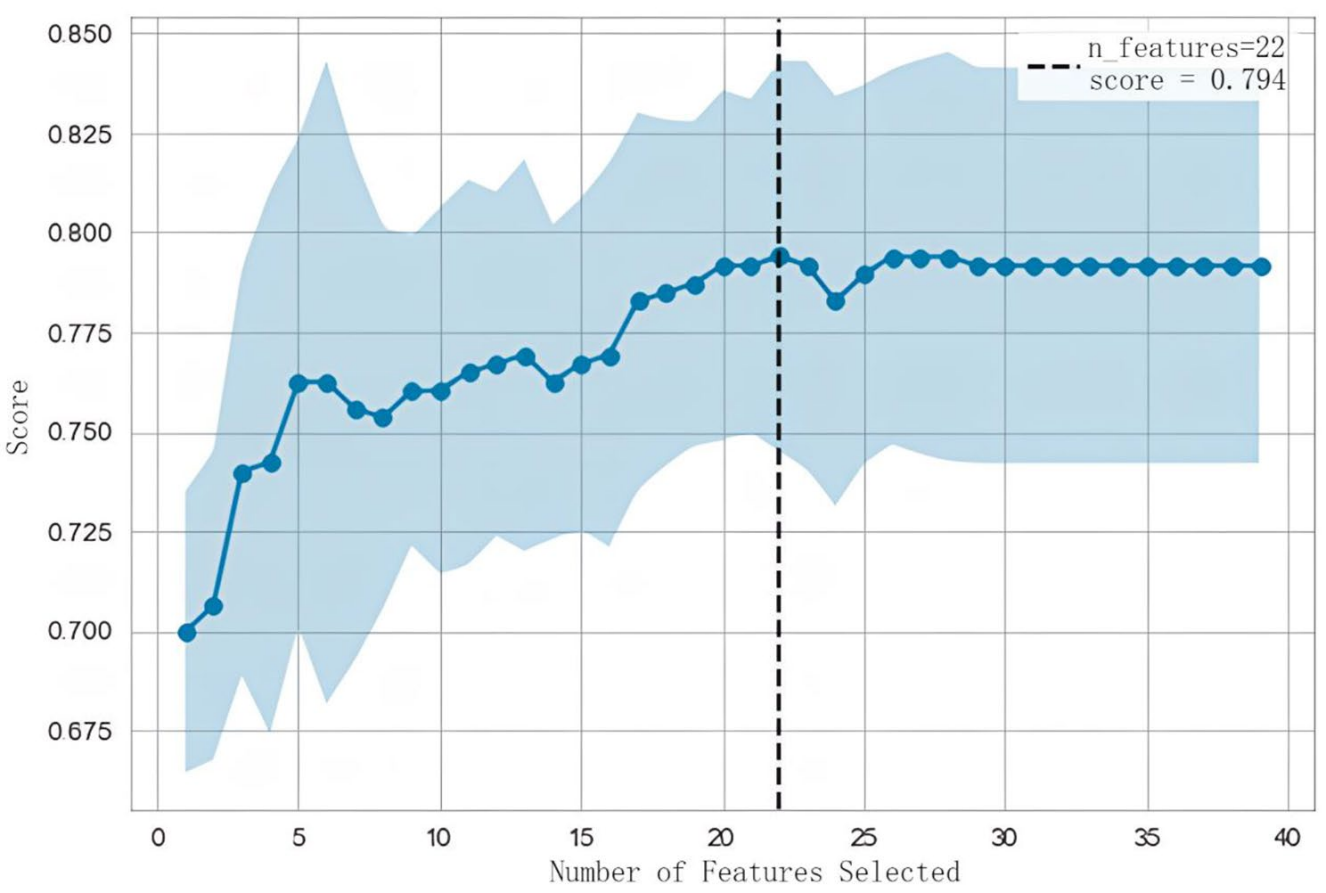
Recursive feature elimination to determine features

Too many features will not only increase the model prediction and training effort, resulting in a decrease in implementation efficiency, but also lead to a more serious overfitting problem. Therefore, on the basis of ensuring performance, in order to ensure a certain degree of generalization ability, this study uses the RFECV algorithm for feature selection, and finally decides to build a model with 22 features, as shown in Fig. 3.

### *Parameter tuning*

The results of tenfold cross-validation of the Adaboost machine learning model with default parameters were shown in Table 3. After tuning the data, the results showed that the average accuracy was 0.7917, AUC was 0.7803, recall was 0.5275, precision was 0.7180, F1 score was 0.5977, Kappa value was 0.4630, and MCC was 0.4793. The classification performance of the 10-fold cross-validation model after parameter optimization was better than the model under the default parameters in terms of accuracy, AUC, and precision. The model under the default parameters and the comparison before and after model tuning were shown in Table 3.

**Table 3:** Evaluation of the learning performance of the adaboost model with default parameters and after optimization of the parameters

10-fold cross- validation	Accuracy	AUC	Recall	Precision	F1-score	Kappa	MCC
Adaboost^a^	0.7894	0.7767	0.5484	0.7056	0.6049	0.4656	0.4799
Adaboost^b^	0.7917	0.7803	0.5275	0.7180	0.5977	0.4630	0.4793

Adaboost^a^ is the model with default parameters

Adaboost ^b^ is the model with optimized parameters

### Effects of the validation set

#### ROC curve

**Fig. 4:**
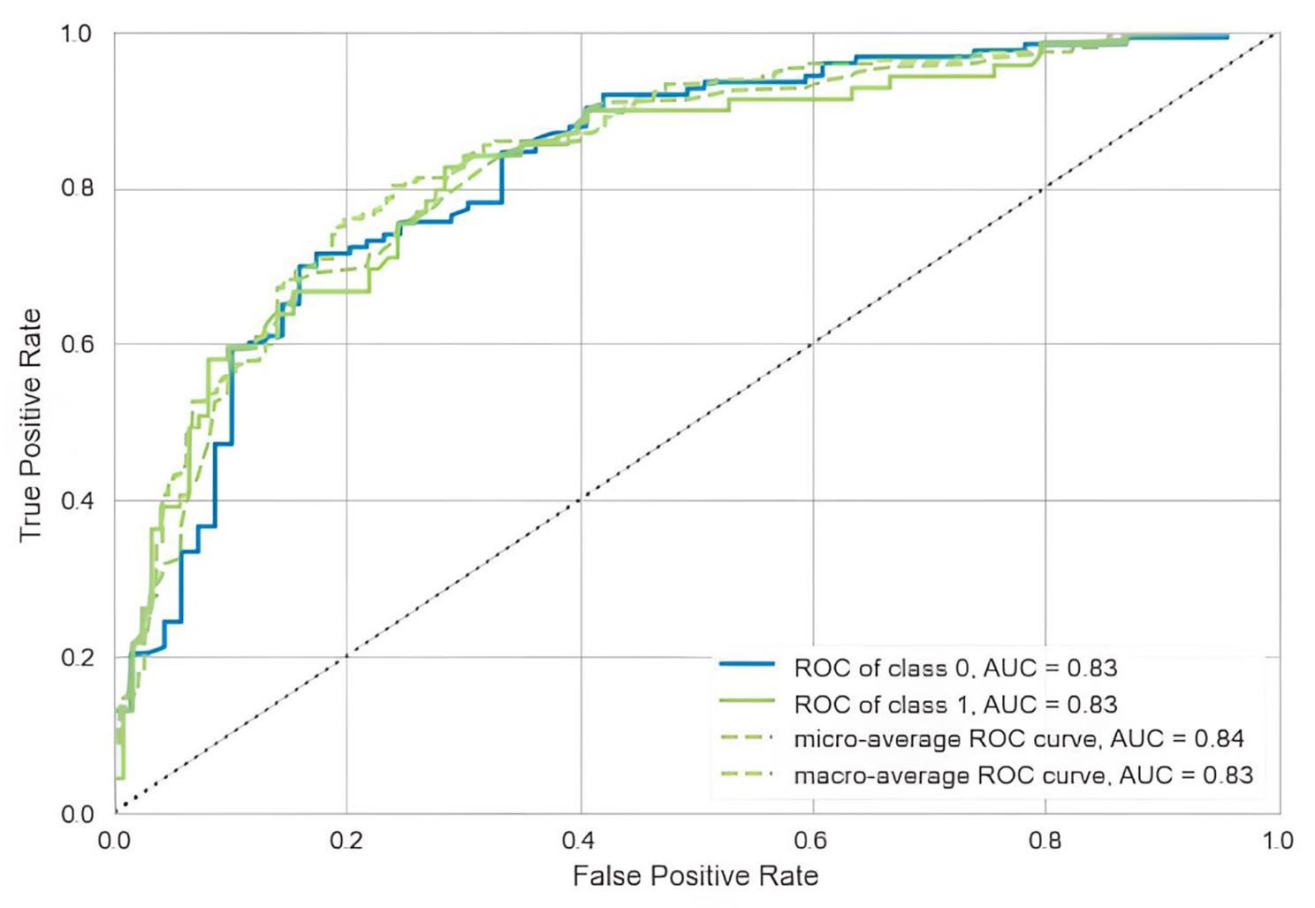
AUC curve of Adaboost model in validation sets

In this study, the Adaboost model was evaluated and the ROC curves of the validation set were plotted. It is generally considered that an AUC greater than 0.7 is a clinically important cut-off, i.e., if the area under the ROC curve of a predictor is greater than 0.7, it can be considered to have a high diagnostic value. In the Adaboost model of this study, the AUC index reached 0.83 as shown in Fig. 4, and the constructed Adaboost model can be considered to have a good discriminative ability.

### *PR curves*

PR curves are most commonly used in class-unbalanced data. The PR curve in our study focuses exclusively on the positive sample, which has a positive to negative ratio of approximately 1:2 and is not a class unbalanced sample.

### *Evaluation of other performances*

**Fig. 5:**
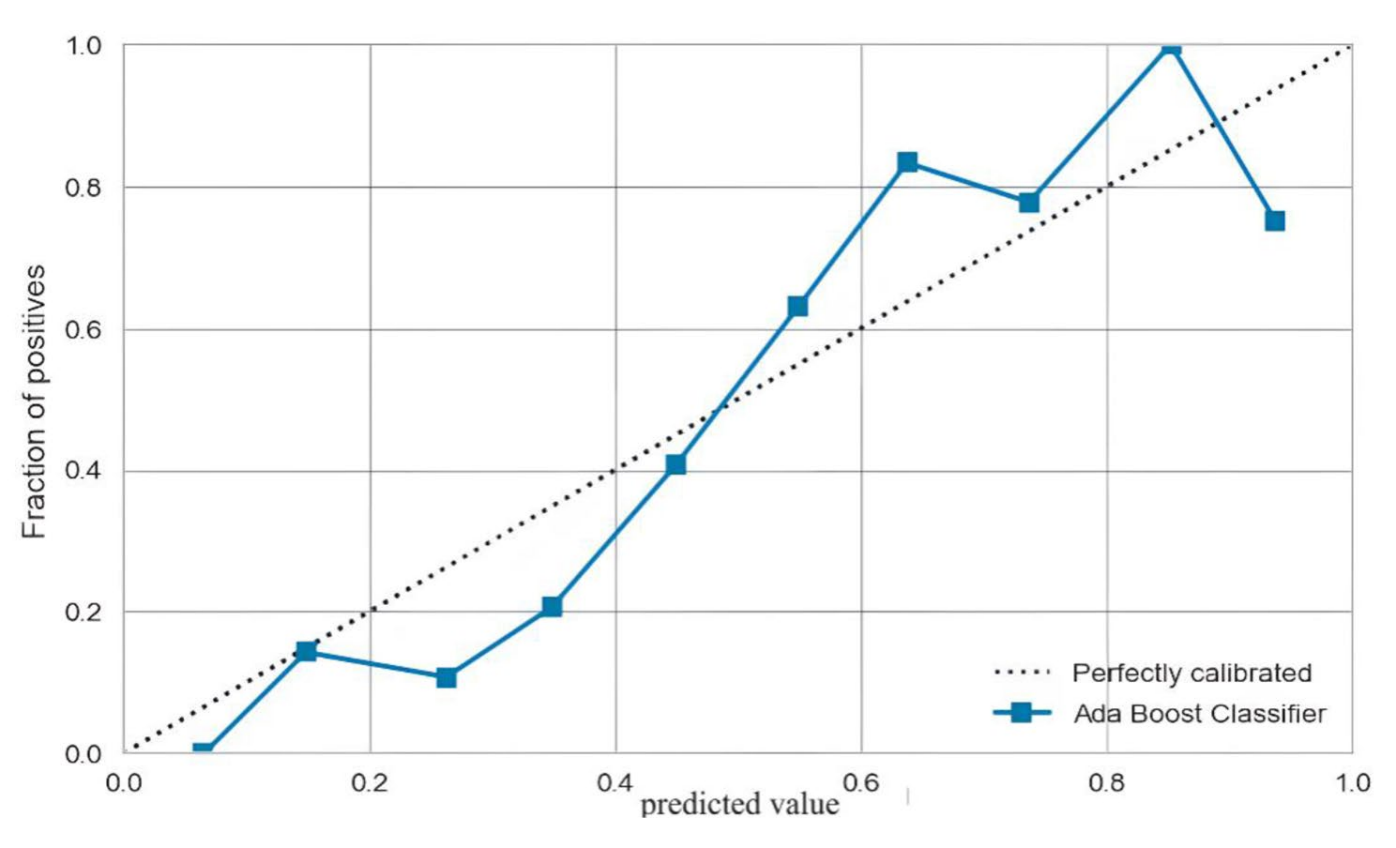
Calibration curves for the Adaboost model

In our study, the accuracy of the validation set reaches 0.7708, the recall reaches 0.4928, the precision reaches 0.7909, the F1 score reaches 0.6071, the Kappa coefficient reaches 0.4574, and the MCC reaches 0.4829, see Table 4. The calibration curve of the validation set is slightly skewed, but the overall calibration effect is still very good, see Fig. 5. In the validation set, the AUC reached 0.83, which verified the excellent classification effect of the model. In conclusion, the Adaboost model constructed in this study not only had a good differentiation, but also had an excellent generalization ability.

**Table 4:** Validation set metrics of Adaboost model algorithm

Model	Accuracy	AUC	Recall	Precision	F1-score	Kappa	MCC
Adaboost	0.7708	0.8273	0.4928	0.7907	0.6071	0.4574	0.4829

## Discussions

This study assessed depression levels in home-quarantined individuals during the COVID-19 epidemic. After a large-scale outbreak of COVID-19 in Shanghai, China, in the first half of 2022, the local government implemented a city closure measure for more than 2 months and required citizens to be home-quarantined. With the economic, social and life changes after the city closure, the home-quarantined people were also affected to different degrees in terms of economy, health, life and work, and some of them experienced various psychological symptoms, such as increased stress, anxiety and depression [2, 29]. The present study showed that the prevalence of depressive symptoms among home-quarantined people during the COVID-19 epidemic was 31.66%.

Based on the psychoemotional changes in the home-quarantined population after the COVID-19 epidemic, the sociodemographic characteristics of this population, factors related to the epidemic, and issues related to the concerns of those who were home-quarantined after the sealing of the city, we conducted a cross-sectional network survey of the population. The aim was to identify and predict the level of depression that occurred in these population after the epidemic, in order to provide a basis for the development of appropriate prevention and control strategies in the future, and also to serve as a reference for the assessment of the negative psychological effects that will occur in the population after the occurrence of similar public health emergencies in the future.

Currently, clinical studies have been conducted to predict depression in patients with various diseases [18–23], while the prediction of depression in people quarantined during epidemics is rare. Some researchers have used machine learning algorithms to predict the psychological status of some populations such as healthcare workers, students, and pregnant women during epidemics [20, 27–28], but depression prediction for home-quarantined people has not been reported from Pubmed searches. In view of this, this study used a machine learning algorithm to predict the depression status of home-quarantined people during the COVID-19 epidemic. In this study, a machine learning algorithm with optimal performance was selected from 15 machine learning algorithms for predicting depression in a home-quarantined population during the COVID-19 epidemic. The Adaboost algorithm selected in this study was superior to the other 14 algorithms in terms of machine learning performance, and this algorithm had not previously been used to predict depression in this population.

Adaboost is an iterative algorithm where the core idea is to train different classifiers (weak classifiers) for the same training set and then aggregate these Adaboost weak classifiers to form a stronger final classifier (strong classifier) [35–36]. The algorithm itself is implemented by changing the data distribution, which determines the weights of each sample based on whether it is correctly classified in each training set and whether it affects the accuracy of the final overall classification. The new data set with modified weights is sent to the lower classifiers for training, and finally the classifiers obtained from each training are fused together as the final decision classifier. The main advantages of the Adaboost algorithm are: (1) when Adaboost is used as a classifier, the classification accuracy is very high; (2) within the framework of Adaboost, it is possible to use a variety of regression classification models to construct weak learners, which is very flexible; (3) when used as a simple binary classifier, the construction is simple and the results are understandable; (4) overfitting does not easily occur.

Adaboost, originally proposed by Freund and Schapire [37], is an acronym for Adaptive Boosting, which is currently the most widely used boosting method [38–40] and has been widely applied in various fields of medicine, such as face recognition, laboratory testing, screening, disease prediction, diagnosis, and treatment [39–52]. Morra et al. [41] used hierarchical Adaboost to diagnose Alzheimer’s disease through automated hippocampal segmentation. Cao et al. [42] and Ghimire et al. [43] used Adaboost for automatic image sentiment classification and facial expression recognition, respectively. Uc-Cetina et al. [44] used Adaboost to automatically detect Chagas parasites in blood images, achieving high accuracy with 100% sensitivity and 93.25% specificity. Hrdlicka et al. [45] used Adaboost to predict schizophrenia with high specificity. Jiménez-García et al. [46] used Adaboost to assess sleep apnea-hypopnea syndrome in children using airflow and oximetry signals. Li et al. [47] used Adaboost’s multi-wavelength spatial frequency domain imaging (SFDI) and characterization to differentiate between abnormal and normal colorectal tissue, which is expected to improve screening in the distal gastrointestinal tract in the future. Hu et al. [48] used an Adaboost classifier combined with EEG features to automatically screen for driver fatigue. Kwon et al. [49] used Adaboost and other methods to screen for osteoporosis in Korean postmenopausal women. Ochs et al. [50] used Adaboost to automatically classify lung bronchovascular anatomy in CT images. Chen et al. [51] reported that the AdaBoost algorithm showed excellent performance in a diabetes classification model based on clinical data. Hao et al. [52] used a modified Adaboost algorithm to detect lung cancer based on electronic nose. Wang et al. [39] used a deep VGG-16 AdaBoost hybrid classifier to analyze the clinical value of combined vaginal ultrasound, magnetic resonance dispersion-weighted imaging, and multi-layer spiral CT for the diagnosis of endometrial cancer. Park et al. [40] reported that Adaboost is useful for ophthalmic small-incision lenticule extraction (SMILE), a surgical procedure for refractive correction of myopia and astigmatism. Adaboost can accurately predict the spherical, cylindrical, and astigmatic axis nomograms for SMILE using a machine learning algorithm. However, although Adaboost is widely used clinically, there have been no reports on predictive modeling of depression in home-bound people during the COVID-19 epidemic. We conducted a study on depression in home-quarantined people during the large-scale COVID-19 epidemic in Shanghai in the first half of 2022. It is a new attempt to apply the Adaboost modeling algorithm to construct a predictive model of depression in this population and achieved satisfactory results, and it is hoped that this model algorithm can be used in the future for depression screening, identification and determination of people at high risk of depression in quarantined populations after disease outbreaks or other emergencies, as well as for taking targeted measures to intervene in the occurrence of the disease.

In this study, the performance of 15 machine learning models was comprehensively evaluated under the default parameters, and the result showed that the Adaboost model had more excellent classification ability in terms of accuracy, F1 score, Kappa and MCC than the other models, and its AUC, recall and precision were also still excellent. After parameter optimization and tenfold cross-validation, the classification effect of this model is more excellent than that of the model with default parameters in terms of accuracy, AUC, and precision. It is generally believed that if the area under the ROC curve of a predictor is greater than 0.7, it can be considered to have a high diagnostic value. In this study, when evaluating of the Adaboost model, the ROC curves of the validation set were plotted, and the AUC index reached 0.83. which can be considered that the Adaboost model constructed in this study also has a good discrimination and excellent generalization ability. In addition, the Kappa coefficient of 0.5630 with moderate consistency in the Adaboost model [53–55] is the highest among the 15 models in our study. The calibration curves were tested in the validation set to evaluate the calibration of the model and still have a good overall calibration.

In our study, the top 6 features in the Adaboost modeling algorithm were ranked in order of importance as nucleic acid positivity, poor mood during quarantine, decreased income, fear of losing one’s job or that of a family member, not being vaccinated, and fear of not being able to do one’s work, respectively. During the COVID-19 epidemic, positive nucleic acid test and not being vaccinated were all related to the prevention and control measures taken, while no income or decreased income, fear of losing one’s job or that of a family member, and fear of not being able to work were related to the economic pressure caused by the implementation of the quarantine measures after the epidemic and the resulting poor mood during the quarantine period. The characteristics we selected in our survey took into account not only sociodemographic characteristics, but also the epidemic, its prevention and control, and the health, living, working, learning, and psychological effects associated with the epidemic. All of these characteristics mentioned above are closely related to the psychological emotions of the quarantined population during the epidemic [56–57]. Therefore, this should be taken into account when using machine learning models to screen and predict depression in relevant populations in the future.

In this study, the Adaboost risk prediction model was constructed and validated by machine learning modeling from multiple dimensions such as sociodemographic characteristics, psychological characteristics, COVID-19 outbreak, and other related factors. In the modeling process, the Adaboost model comprehensively considered the various influencing factors of depression in home-quarantined individuals and gave the importance of the characteristic factors with high prediction accuracy. The Adaboost algorithm constructed in our study had great importance in detecting the depressive symptoms of subjects, which can help to screen and identify the relevant populations in case of COVID-19 epidemic or public health emergency, identify high-risk populations, provide targeted interventions for them, After finding and identifying these high-risk groups, psychologists or counselors can communicate with quarantined people via phone or WeChat to reduce their psychological burden, eliminate or alleviate the factors that lead to depression, control their depressive symptoms, and enable them to return to a healthy state as soon as possible. Meanwhile, during the period of quarantine, ways to reduce or eliminate the quarantined person’s depression can also be publicized through television and the Internet. For example, depressed home-quarantined people stay at home for a long time and sometimes receive too much information about the epidemic and its dangers from the Internet and other media [58–59], which has a negative effect on them; at the same time, because quarantined people are at home, they have fewer opportunities to communicate with other people face to face, which increases their chances of developing depression. Therefore, it is important to explain to people in quarantine that they should not seek more information about the epidemic and its dangers and should not receive too much negative information.

### Limitations

The disadvantages of this study were as follows: first, this study used a web-based cross-sectional survey to avoid face-to-face contact and its possible infections, and the sample selection may be biased and not representative enough; second, The Adaboost algorithm used in our study was only applicable to the prediction of depression symptoms and precipitating factors in home-quarantined individuals during the COVID-19 epidemic, but not to the depression status of other populations during the non-epidemic period; third, some important characteristics may not have been included in the Adaboost prediction model for home-quarantined people during the COVID-19 epidemic, which should be considered in future applications of the model.

## Conclusion

In conclusion, in this study, we conducted a cross-sectional survey on the depression status of home-quarantined subjects during the COVID-19 epidemic in Shanghai in the first half of 2022. We found that the prevalence of depressive symptoms in this population was 31.66%. In addition, the most optimized Adaboost model was selected from 15 machine learning algorithms. The Adaboost model showed good superiority in predicting depression in the home-bound population, suggesting that the depression risk model of home-bound people established by the Adaboost algorithm can effectively predict the depression status of the home-quarantined individuals under COVID-19 mass epidemic conditions, and we are currently in the process of further external validation of the model algorithm; however, the applicability of the algorithm to mental health conditions under the conditions of other infectious disease epidemics awaits further research in the future.

### Electronic supplementary material

Below is the link to the electronic supplementary material.

Supplementary Material 1

## Data Availability

﻿The datasets used and analyzed in this study are available from the corresponding author on reasonable request. Because of the sensitive nature of the data collected on the mental health of home-quarantined residents amongst which individuals are potentially identifable, we cannot provide open access to our data.
