# Supplementary material for: Construction and validation of machine learning algorithm for predicting depression among home-quarantined individuals during the large-scale COVID-19 outbreak: based on Adaboost model
Source: BMC Psychol. 2024 Apr 24;12:230. doi: 10.1186/s40359-024-01696-8 (PMC11044386; doi:10.1186/s40359-024-01696-8)
Supplement: Supplementary file 1 — Supplementary Material 1 [file 40359_2024_1696_MOESM1_ESM.docx]

QUESTIONNAIRE

Greetings! In order to understand the impact of COVID-19 on the mental health of individuals in home quarantine, we have conducted this network survey and would like to obtain your cooperation. You only need to answer according to your own experience. Your answers will be kept strictly confidential and will not be shared with others, and you give your informed consent. If you do not wish to answer, please do not complete the questionnaire. Thank you for your assistance.

1. Gender

Male female

1. Age

≤19 20-29 30-39 40-49 50-59 ≥60

1. Occupations

Architecture & Engineering

Business & Finance

IT & Computing

Art & Design

Medicine & Health

Education

Science & Research

Administration & Justice

Media & Communications

Food & Nutrition

Agriculture, Forestry & Other

1. Professional title

Nor or Junior Middle Senior

1. Married

Yes No

1. Income monthly(RMB)

≤2000 2000-4999 5000-9999 ≥10000

1. Date of start of quarantine

Date: day month

1. Nucleic acid testing

Positive Negative

1. How much do you know about the COVID-19 vaccine?

Nor or a little Some Very well

9. whether you have been vaccinated?

Yes No

14.Are you concerned about the following (multiple choices)

COVID-19 Yes No

Health of family members Yes No

Employment for yourself or your children Yes No

Children's schooling Yes No

Daily life of the family Yes No

Traffic halt Yes No

Lack of daily necessities during quarantine Yes No

Lack of foods during quarantine Yes No

Missing family and friends Yes No

Feeling lonely Yes No

Fear of losing your job Yes No

Fear of spreading the disease to others Yes No

Fear of nor or decrease income Yes No

Worry about inconvenience in life Yes No

Feeling inconvenienced by family and society Yes No

Do not understand the existing quarantine measures Yes No

Dissatisfied with the existing epidemic prevention measures Yes No

Worried about the cost of treatment Yes No

Feeling of inconvenience to family and society Yes No

Lack of understanding of existing quarantine measures Yes No

Dissatisfaction with the effectiveness of existing prevention and control measures Yes No

Worried about the cost of treatment Yes No

PATIENT HEALTH QUESTIONNAIRE (PHQ-9)

| Over the last 2 weeks, how often have you been bothered by any of the following problems?  (use "○" to indicate your answer) | Not at all | Several days | More than half the days | Nearly every day |
| --- | --- | --- | --- | --- |
| 1. Little interest or pleasure in doing things | 0 | 1 | 2 | 3 |
| 2. Feeling down, depressed, or hopeless | 0 | 1 | 2 | 3 |
| 3. Trouble falling or staying asleep, or sleeping too much | 0 | 1 | 2 | 3 |
| 4. Feeling tired or having little energy | 0 | 1 | 2 | 3 |
| 5. Poor appetite or overeating | 0 | 1 | 2 | 3 |
| 6.Feeling bad about yourself or that you are a failure or have let yourself or your family down | 0 | 1 | 2 | 3 |
| 7.Trouble concentrating on things, such as reading the newspaper or watching television | 0 | 1 | 2 | 3 |
| 8.Moving or speaking so slowly that other people could have noticed. Or the opposite being so fidgety or restless that you have been moving around a lot more than usual | 0 | 1 | 2 | 3 |
| 9. Thoughts that you would be better off dead, or of hurting yourself | 0 | 1 | 2 | 3 |
